# Interplay between Perovskite Magic-Sized Clusters and Amino Lead Halide Molecular Clusters

**DOI:** 10.34133/2021/6047971

**Published:** 2021-01-07

**Authors:** Evan T. Vickers, Ziyi Chen, Vivien Cherrette, Tyler Smart, Peng Zhang, Yuan Ping, Jin Z. Zhang

**Affiliations:** ^1^Department of Chemistry and Biochemistry, University of California, Santa Cruz, CA 95064, USA; ^2^Department of Chemistry, Dalhousie University, Halifax, NS, Canada B3H 4R2; ^3^Department of Physics, University of California, Santa Cruz, CA 95064, USA

## Abstract

Recent progress has been made on the synthesis and characterization of metal halide perovskite magic-sized clusters (PMSCs) with *ABX*_3_ composition (*A* = CH_3_NH_3_^+^ or Cs^+^, *B* = Pb^2+^, and *X* = Cl^−^, Br^−^, or I^−^). However, their mechanism of growth and structure is still not well understood. In our effort to understand their structure and growth, we discovered that a new species can be formed without the CH_3_NH_3_^+^ component, which we name as molecular clusters (MCs). Specifically, CH_3_NH_3_PbBr_3_ PMSCs, with a characteristic absorption peak at 424 nm, are synthesized using PbBr_2_ and CH_3_NH_3_Br as precursors and butylamine (BTYA) and valeric acid (VA) as ligands, while MCs, with an absorption peak at 402 nm, are synthesized using solely PbBr_2_ and BTYA, without CH_3_NH_3_Br. Interestingly, PMSCs are converted spontaneously overtime into MCs. An isosbestic point in their electronic absorption spectra indicates a direct interplay between the PMSCs and MCs. Therefore, we suggest that the MCs are precursors to the PMSCs. From spectroscopic and extended X-ray absorption fine structure (EXAFS) results, we propose some tentative structural models for the MCs. The discovery of the MCs is critical to understanding the growth of PMSCs as well as larger perovskite quantum dots (PQDs) or nanocrystals (PNCs).

## 1. Introduction

Semiconductor perovskite nanocrystals (PNCs) or quantum dots (PQDs) based on the formula *ABX*_3_ (*A* = CH_3_NH_3_^+^ (MA^+^), CH(NH_2_)_2_^+^ (FA) or Cs^+^; *B* = Pb^2+^, Sn^2+^; *X* = Cl^−^, Br^−^, or I^−^) have drawn significant research interests due to their bandgap tunability, high photoluminescence quantum yield (PLQY), and sharp emission peaks that are attractive for LEDs and other optoelectronic applications [1–12]. However, there is limited knowledge about the structure and growth mechanism at the molecular or atomic level [13–15].

Perovskite magic-sized clusters (PMSCs) are particles with a single size or narrow size distribution that are smaller than PNCs but still contain the same perovskite composition [16–21]. In addition, PMSCs possess unique optical properties compared to PNCs such as bluer and sharper absorption and emission bands [22, 23]. They are also important for understanding the underlying mechanisms of perovskite crystal growth [24–35]. However, one major challenge in the study of PMSCs is to determine their structure. Given the ultrasmall size of PMSCs, PMSCs do not have a long enough ordered structure to be characterized by X-ray diffraction and cannot be detected by imaging techniques, such as transmission electron microscopy (TEM). Moreover, due to their instability in polar solvents, PMSCs cannot be measured by a mass spectrometry. Thus far, the only evidence of their presence is their characteristic optical absorption and emission bands, which are sharp, well-defined, and significantly blue-shifted with respect to their bulk form [16–21].

In our effort to better understand the structure and growth process of PMSCs using CH_3_NH_3_PbBr_3_ as an example, we made an important discovery in one of our control experiments that a new species could be generated, without the CH_3_NH_3_^+^ or methylammonium (MA^+^), that absorbs bluer (402 nm) than the PMSCs (424 nm) with BTYA and VA as ligands. This species clearly does not have the full perovskite composition since MA^+^ is not present. Therefore, we attribute this new species to be a MC that contains the components Pb^2+^, Br^−^, and BTYA. We carried out a more detailed study to help shed light on these MCs and their relation to PMSCs. In addition, the MC synthesis was demonstrated not only using PbBr_2_ but also was successful with PbCl_2_ and PbI_2_ precursors. Furthermore, extended X-ray absorption fine structure (EXAFS) studies were conducted to help gain some understanding of their structural compositions.

## 2. Results and Discussion

**Figure 1:**
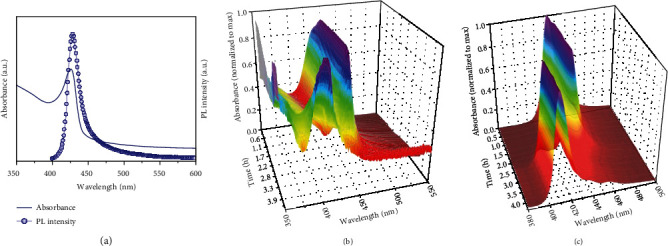
Initial UV-Vis absorption and PL spectra with 365 nm excitation (a). The absorption (b) and PL (c) spectra overtime of MAPbBr_3_-VA-BTYA PMSCs.

MAPbBr_3_ PMSCs with BTYA and VA passivating ligands were synthesized using the reprecipitation method, as described in our previous report [20]. Detail of the MAPbBr_3_-VA-BTYA PMSC synthesis is described in the supporting information (SI). The UV-vis absorption and PL spectra of MAPbBr_3_-VA-BTYA PMSC solution are shown in Figure 1(a), with the major absorption peak at 424 nm and PL peak at 434 nm, as we reported previously [20]. Interestingly, these optical peaks decrease in intensity, and simultaneously, a new absorption peak at 402 nm and PL peak at 410 nm appear with intensity increasing over time. Figure 1(b) shows the change of the absorption spectra overtime. The absorption peak initially increases in intensity and red shifts from 424 nm to 432 nm, indicating an initial growth of the PMSCs. After ~14 minutes, the 432 nm peak decreases in intensity while a new peak at 402 nm appears and increases in intensity. After ~4.3 h, the 432 nm peak completely disappears, leaving behind the 402 nm peak with intensity similar to that of the original 424 nm peak, and no change is observed thereafter.

Similar observations are made in the PL spectra, as shown in Figure 1(c). The 434 nm PL peak increases in intensity in the first ~14 minutes, and then begins to decrease. As the 434 nm PL peak's intensity decreases, a new 410 nm PL peak appears and increases in intensity until the complete disappearance of the 434 nm PL peak. The changes in both the absorption and PL spectra are observed with an isosbestic point at 419 and 425 nm, respectively. This suggests that there is an equilibrium between the two species, and that they are mechanistically related. This phenomenon has been previously reported for CdSe nanoparticles [36]. When an excess amount of BTYA was added to a ~1.6 nm CdSe nanoparticle solution, a broad absorption band centered at 445 nm was converted into a very narrow absorption band which centered at 414 nm. In this previous report, Cd-Se bonds are broken until a thermodynamically stable size or structure configurates. Similarly, in the present work, Pb-Br bonds in the PMSCs may break following similar principles as the CdSe nanoparticles. In our previous study, we suggested that the 402 nm absorption peak and 410 nm PL peak are derived from smaller PMSCs that fragmented from a larger PMSC. However, in the present study, we found through control experiments that the 402 nm peak is not due to PMSCs but can be attributed to molecular clusters (MCs), as explained next.

**Figure 2:**
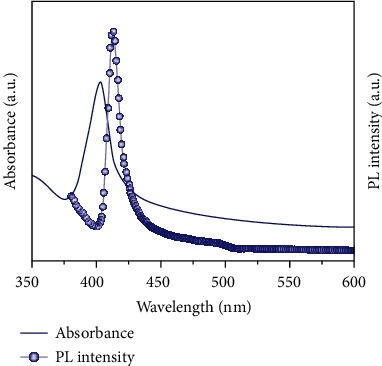
UV-Vis absorption and PL spectra of PbBr_2_-VA-BTYA.

In order to elucidate the structure and growth mechanism of this new species absorbing at 402 nm, labeled as MC402, we designed and conducted a set of control experiments. First, we investigated the effect of performing the synthesis without the *A* component (MA^+^) in the *ABX*_3_ perovskite. We performed the same reprecipitation reaction using only PbBr_2_, VA, and BTYA precursor components. Detail of the PbBr_2_-VA-BTYA synthesis is described in the SI. As shown in Figure 2, a sharp and strong absorption band peaked at 402 nm is observed, along with a PL peak at 410 nm. The PLQY of the PbBr_2_-VA-BTYA sample is determined to be 23 ± 5% (vs quinine sulfate, 58%), which is slightly higher than the previously reported MAPbBr_3_-VA-BTYA PMSCs (19 ± 3%). The FWHM of the absorption band is 11 nm, while that of the PL band is 10 nm. Compared to MAPbBr_3_-VA-BTYA PMSCs at their initial optical spectra, these bandwidths are significantly narrower. However, their peak positions as well as their bandwidths are very similar or the same as that of the MAPbBr_3_-VA-BTYA PMSCs after they are transformed into the new species absorbing at 402 nm with PL at 410 nm. This indicates that the new species transformed from the original MAPbBr_3_-VA-BTYA PMSCs is the same as the product synthesized using PbBr_2_-VA-BTYA without the MA. At this point, because VA protonates BTYA to produce BTYA^+^, we assumed that BTYA^+^ could be playing the role of the *A* cation in the *ABX*_3_ perovskite structure. However, further experiments show that this is not the case. The bluer absorption and PL peaks of the MCs compared to PMSCs are largely due to their smaller size or more localized electronic wave functions, essentially quantum confinement.

**Figure 3:**
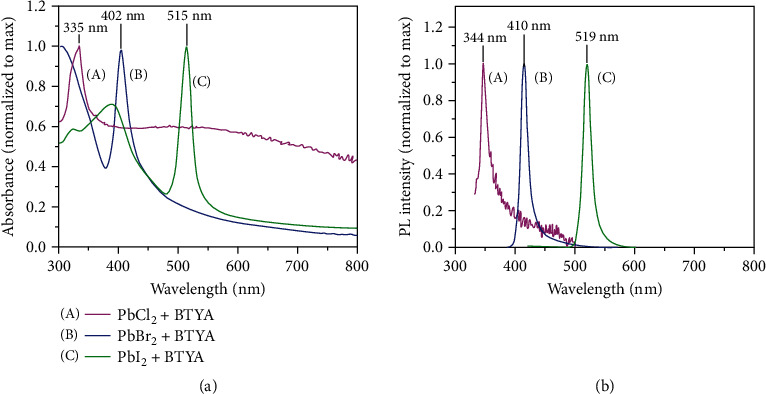
UV-Vis absorption (a) and PL (b) spectra of PbX_2_-BTYA.

As another important control experiment, only PbBr_2_ and BTYA components were added to the precursor solution and reprecipitated, following the same method of synthesis. In this case, without the VA component, BTYA cannot be protonated, and therefore, there is no *A* cation component present in the precursor solution. Interestingly and surprisingly, the PbBr_2_-BTYA sample exhibits the same characteristic optical spectra as PbBr_2_-VA-BTYA or the final transformed product of MAPbBr_3_-VA-BTYA PMSCs. In addition, we extended the synthesis from PbBr_2_ to PbCl_2_ and PbI_2_ as the precursor to mix with BTYA. Their UV-vis absorption and PL spectra of the synthesized products are shown in Figures 3(a) and 3(b). The absorption peaks are 335, 402, and 515 nm, while the PL bands are centered at 344, 410, and 519 nm for PbCl_2_-BTYA, PbBr_2_-BTYA, and PbI_2_-BTYA samples, respectively. The dependence of the peak position on the halide is evidence that the halide is part of the structure of the MCs. The FWHM of their absorption bands is 18, 15, and 13 nm, respectively, while the FWHM of their PL bands is all around 10 nm. These significantly blue-shifted peak positions, with respect to their perovskite bulk form or NCs, and very narrow optical bandwidths indicate the presence of ultrasmall and well-defined clusters for each PbX_2_-BTYA sample. For PbCl_2_-BTYA, in addition to the sharp absorption band peaked at 335 nm, there is a broad background that is likely due to larger aggregated structures. While the PLQY of PbCl_2_-BTYA is very low (>0.1%), indicating a high density of trap states, the PLQY for PbBr_2_-BTYA and PbI_2_-BTYA is much higher at 26 ± 6% and 11 ± 3%, respectively.

The interesting question is how the different samples, MAPbBr_3_-VA-BTYA, PbBr_2_-VA-BTYA, and PbBr_2_-BTYA, all show the same 402 nm absorption and 410 nm PL bands? Without the *A* cation component for PbBr_2_-VA-BTYA, and without ammonium cation for PbBr_2_-BTYA, we can only attribute the species responsible for these optical bands to molecular clusters (MCs) rather than PMSCs since they clearly do not have the full *ABX*_3_ composition. Because their optical properties only depend on and originate from the Pb^2+^, Br^−^, and BTYA components, the PbBr_2_-BTYA MCs are thermodynamically more stable since they can form even when methylammonium ions are present to compete to form PMSCs.

To confirm the presence and role of the BTYA ligand, FTIR spectra were collected for PbCl_2_-BTYA, PbBr_2_-BTYA, and PbI_2_-BTYA MCs as well as free unbound BTYA ligands. As shown in Figure S1, there is a shift in N-H_2_ bending frequency from free ligand at 1606 cm^−1^ to 1586, 1579, and 1571 cm^−1^ for PbCl_2_-BTYA, PbBr_2_-BTYA, and PbI_2_-BTYA MCs, respectively. This is consistent with shift to lower frequency when ligand is bound to heavier atoms [37, 38]. The N-H_2_ bending peak for PbCl_2_-BTYA, PbBr_2_-BTYA, and PbI_2_-BTYA MCs is sharp and narrow, while the N-H_2_ bending for BTYA is broader. This may indicate that BTYA ligands are more ordered when attached to a MC, while the free unbound BTYA ligands are in a more inhomogeneous environment. Furthermore, the N-H_2_ bending peak for the PbX_2_-BTYA MCs is symmetrical and only contains a single peak, indicating a highly homogeneous environment for the BTYA ligands on the PbX_2_-BTYA MC surface. This is also evidence that the MCs are highly uniform or have a single or narrow size distribution. In addition, the N-H aliphatic primary amine stretch at 3369 and 3288 cm^−1^ present for the free ligands is absent for the PbX_2_-BTYA MCs. However, for PbCl_2_-BTYA, PbBr_2_-BTYA, and PbI_2_-BTYA MCs, a sharp peak at 3515, 3511, and 3495 cm^−1^, respectively, is present, corresponding to the N-H stretch in lead(II)-halide-butylamide (PbX*_n_*[NH(CH_2_)CH_3_]). Overall, the FTIR spectra confirms that BTYA interacts with the MC surface and supplies further evidence of the presence of well-defined and discretely sized MCs.

**Figure 4:**
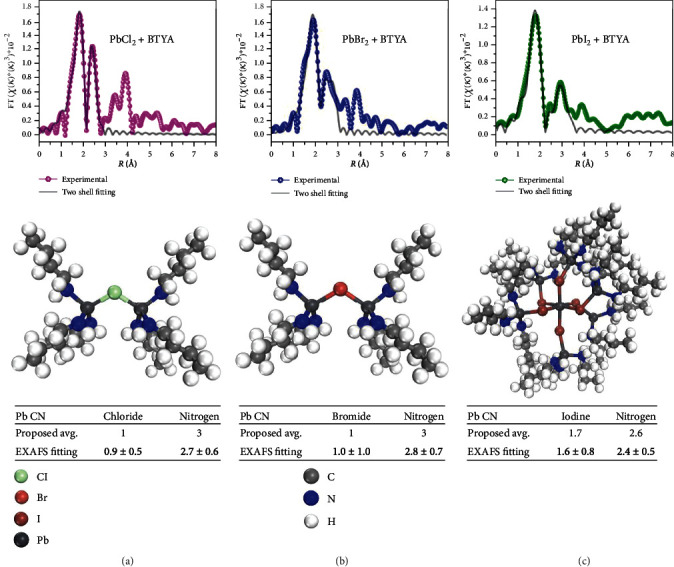
FT-EXAFS spectra, molecular models, and the Pb CN from the proposed model and EXAFS fitting results for (a) PbCl_2_-BTYA, (b) PbBr_2_-BTYA, and (c) PbI_2_-BTYA MCs.

To elucidate the structure of PbX_2_-BTYA MCs, X-ray absorption spectroscopy measurement was conducted on the Pb L(II) edge, which corresponds to the electron transition from 2*p*_1/2_ to unoccupied *d* states [39–41]. Further detail of the measurement is in the SI. The Fourier transformed EXAFS (FT-EXAFS) spectra of PbCl_2_-BTYA, PbBr_2_-BTYA, and PbI_2_-BTYA MCs are shown in Figures 4(a)–4(c), and Table 1 summarizes the coordination number (CN) and bond distance (*R*) of Pb-N and Pb-X bonding from the two-shell fitting results. For each sample, the highest intensity peak corresponds to the Pb-N bonding, providing evidence for the BTYA coordination to Pb^2+^ via the lone pair electrons on the amine functional group. The higher Pb-N coordination than Pb-X provides further evidence that the samples represent MCs rather than an ordered perovskite structure. In addition, due to the low Pb-X coordination, the MCs must be positively charged, as the simplest possible structure is 3+ and becomes increasingly positive as the proposed structure increases in size.

The simplest proposed model and most closely matched bonding coordination number from an EXAFS two shell fitting for PbCl_2_-BTYA and PbBr_2_-BTYA MCs is shown in Figures 4(a) and 4(b). Two Pb^2+^ atoms are bridged by a halogen anion, while the Pb^2+^ atoms are also coordinated with 3 BTYA molecules via nitrogen's lone electron pair. Given the relatively large uncertainty in coordination number and the numerous potential coordination numbers of Pb^2+^, an exact model is not possible at this point. However, the real structure should be similar or close to what we have proposed. For PbI_2_-BTYA MCs, the Pb-I coordination is slightly higher at 1.6 ± 0.8 compared to PbCl_2_-BTYA and PbBr_2_-BTYA MCs at 0.9 ± 0.5 and 1.0 ± 1.0, respectively. Therefore, a different model for PbI_2_-BTYA MCs is proposed to match the coordination more directly from the EXAFS fitting, as shown in Figure 4(c). We propose that a PbI_6_ is present in the sample. In addition, the I^−^ anion bridges an additional Pb^2+^ atom that is terminated by 3 BTYA molecules.

**Figure 5:**
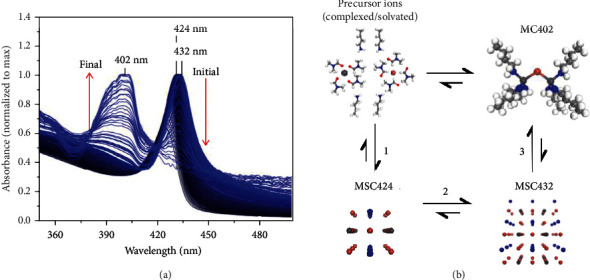
(a) Change of UV-Vis absorption spectra during the transformation from MAPbBr_3_-VA-BTYA PMSCs to PbBr_2_-BTYA MCs and (b) a scheme illustrating the relation among precursor ions, MCs, and MSCs.

From these structural studies, we illustrate in Figures 5(a) and 5(b) the relationship among the MAPbBr_3_-VA-BTYA PMSCs, PbBr_2_-BTYA MCs, and the initial precursor ions that are solvated or complexed with ligands in solution. In Figure 5(a), as indicated by the red shift in the absorption spectra from 424 to 432 nm, the MAPbBr_3_-VA-BTYA PMSCs initially grow in size. The PMSC structure likely rearranges itself to accommodate for BTYA complexation. The restructured intermediate with an absorption peak at 432 nm finally transforms into the MCs. As shown in Figure 5(b), our results suggest two pathways to forming MCs. The first is direct formation from the precursor ions, with no *A* component or ammonium ions. The second pathway is to start with precursor ions, form MSC424, convert into MSC432, and finally form MCs. Under the experimental conditions we have explored so far, we observed clear evidence for transformation from PMSCs to MCs, but not from MCs to PMSCs, indicating that MCs are more stable than PMSCs under the current conditions.

To investigate reversibility from MCs back to PMSCs, we added additional MABr to push the equilibrium back to PMSCs. As shown in Figure S2, the UV-vis absorption spectra were measured after the PMSCs were converted to MCs and with the addition of MABr. As indicated by the absorption spectra, after the addition of MABr, MCs immediately began to convert back to PMSCs. Moreover, as the reaction temperature was decreased from 100°C to 15°C, the equilibrium increasingly favors the formation of PMSCs. This demonstration of reversibility also indicates that these MCs are smaller and more stable than the related PMSCs.

## 3. Conclusion

In summary, we have made an interesting finding on the transformation from MAPbBr_3_-VA-BTYA PMSCs to PbBr_2_-BTYA MCs. This is confirmed by monitoring the MAPbBr_3_-VA-BTYA PMSCs absorption spectra overtime. When the absorption band centered at 424 nm progressively transformed to 402 nm, there is an isosbestic point, suggesting that an equilibrium exist between the two absorption bands and species. After probing the synthesis and transformation through a series of control experiments, we conclude that the absorption band centered at 402 nm derived from only the Pb^2+^, Br^−^, and BTYA components is from MCs rather than PMSCs, because MA^+^ is absent. In addition, we demonstrated that this synthesis can be extended also to PbCl_2_ and PbI_2_ precursors. EXAFS studies confirm that these new species are MCs rather than PMSCs and help to established structural models. The results lead us to conclude that the Pb-Br bridging bonds in the PMSC structure are broken when smaller and thermodynamically more stable MCs form. This interplay between the PMSC and MCs indicates that the MCs are a possible precursor of the PMSC. The findings from this work are important for understanding the mechanism of growth of both bulk and nanostructured perovskites including PMSCs, PNCs, or PQDs that have great potential for many emerging applications.

## Figures and Tables

**Table 1:** Coordination number (CN) as well as Pb-N and Pb-X bond distances for PbX_2_-BTYA MCs.

	Pb-N	Pb-X
PbCl_2_ + BTYA	CN: 2.70 ± 0.6 *R*: 2.39 ± 0.01 Å	CN: 0.9 ± 0.5 *R*: 2.82 ± 0.01 Å

PbBr_2_ + BTYA	CN: 2.80 ± 0.7 *R*: 2.42 ± 0.02 Å	CN: 1.0 ± 1.0 *R*: 2.99 ± 0.02 Å

PbI_2_ + BTYA	CN: 2.40 ± 0.5 *R*: 2.40 ± 0.01 Å	CN: 1.60 ± 0.8 *R*: 3.16 ± 0.03 Å

## Supplementary Materials

Supplementary Materials Figure S1: PbX2-BTYA FT-IR spectra. Figure S2: conversion of MABr+PbBr2 + VA + BTYA MCs back to PMSCs.

## References

[1] Wei Y., Li X., Chen Y. (2020). In situ light-initiated ligands cross-linking enables efficient all-solution processed perovskite light-emitting diodes. *Journal of Physical Chemistry Letters*.

[2] Ma D., Todorović P., Meshkat S. (2020). Chloride insertion-immobilization enables bright, narrowband, and stable blue-emitting perovskite diodes. *Journal of the American Chemical Society*.

[3] Zou C., Liu Y., Ginger D. S., Lin L. Y. (2020). Suppressing efficiency roll-off at high current densities for ultra-bright green perovskite light-emitting diodes. *ACS Nano*.

[4] Vickers E. T., Enlow E. E., Delmas W. G. (2020). Enhancing charge carrier delocalization in Perovskite quantum dot solids with energetically aligned conjugated capping ligands. *ACS Energy Letters*.

[5] Yan F., Tan S. T., Li X., Demir H. V. (2019). Light generation in lead halide perovskite nanocrystals: LEDs, color converters, lasers, and other applications. *Small*.

[6] Zhao X., Ng J. D. A., Friend R. H., Tan Z.-K. (2018). Opportunities and challenges in perovskite light-emitting devices. *ACS Photonics*.

[7] Wang Y., Ding G., Mao J.-Y., Zhou Y., Han S.-T. (2020). Recent advances in synthesis and application of perovskite quantum dot based composites for photonics, electronics, and sensors. *Science and Technology of Advanced Materials*.

[8] El-Ballouli A. O., Bakr O. M., Mohammed O. F. (2019). Compositional, processing, and interfacial engineering of nanocrystal- and quantum-dot-based perovskite solar cells. *Chemistry of Materials*.

[9] Vickers E. T., Xu K., Li X., Zhang J. Z. (2020). Dependence of stability and electronic and optical properties of perovskite quantum dots on capping ligand chain length. *The Journal of Chemical Physics*.

[10] Vickers E. T., Graham T. A., Chowdhury A. H. (2018). Improving charge carrier delocalization in perovskite quantum dots by surface passivation with conductive aromatic ligands. *ACS Energy Letters*.

[11] Li C.-H. A., Zhou Z., Vashishtha P., Halpert J. E. (2019). The future is blue (LEDs): why chemistry is the key to perovskite displays. *Chemistry of Materials*.

[12] Chen D., Chen X. (2019). Luminescent perovskite quantum dots: synthesis, microstructures, optical properties and applications. *Journal of Materials Chemistry C*.

[13] Liu C., Cheng Y.-B., Ge Z. (2020). Understanding of perovskite crystal growth and film formation in scalable deposition processes. *Chemical Society Reviews*.

[14] Hu H., Singh M., Wan X., Tang J., Chu C.-W., Li G. (2020). Nucleation and crystal growth control for scalable solution-processed organic-inorganic hybrid perovskite solar cells. *Journal of Materials Chemistry A*.

[15] Nayak P. K., Moore D. T., Wenger B. (2016). Mechanism for rapid growth of organic-inorganic halide perovskite crystals. *Nature Communications*.

[16] Peng L., Dutta A., Xie R., Yang W., Pradhan N. (2018). Dot-wire-platelet-cube: step growth and structural transformations in CsPbBr3 perovskite nanocrystals. *ACS Energy Letters*.

[17] Xu Y., Zhang Q., Lv L. (2017). Synthesis of ultrasmall CsPbBr3nanoclusters and their transformation to highly deep-blue-emitting nanoribbons at room temperature. *Nanoscale*.

[18] Xu K., Vickers E. T., Luo B. (2020). First synthesis of Mn-doped cesium lead bromide perovskite magic sized clusters at room temperature. *Journal of Physical Chemistry Letters*.

[19] Xu K., Vickers E. T., Luo B. (2020). Room temperature synthesis of cesium lead bromide perovskite magic sized clusters with controlled ratio of carboxylic acid and benzylamine capping ligands. *Solar Energy Materials & Solar Cells*.

[20] Vickers E. T., Xu K., Dreskin B. W., Graham T. A., Li X., Zhang J. Z. (2019). Ligand dependent growth and optical properties of hybrid organo-metal halide perovskite magic sized clusters. *Journal of Physical Chemistry C*.

[21] Xu K., Allen A.’. L. C., Luo B. (2019). Tuning from quantum dots to magic sized clusters of CsPbBr_3_ using novel planar ligands based on the trivalent nitrate coordination complex. *Journal of Physical Chemistry Letters*.

[22] Palencia C., Yu K., Boldt K. (2020). The future of colloidal semiconductor Magic-Size clusters. *ACS Nano*.

[23] Harrell S. M., McBride J. R., Rosenthal S. J. (2013). Synthesis of ultrasmall and magic-sized CdSe nanocrystals. *Chemistry of Materials*.

[24] Jiang Z.-J., Kelley D. F. (2010). Role of magic-sized clusters in the synthesis of CdSe nanorods. *ACS Nano*.

[25] Liu Y., Rowell N., Willis M. (2019). Photoluminescent colloidal nanohelices self-assembled from CdSe magic-size clusters via Nanoplatelets. *Journal of Physical Chemistry Letters*.

[26] Zhang B., Zhu T., Ou M. (2018). Thermally-induced reversible structural isomerization in colloidal semiconductor CdS magic-size clusters. *Nature Communications*.

[27] Nevers D. R., Williamson C. B., Hanrath T., Robinson R. D. (2017). Surface chemistry of cadmium sulfide magic-sized clusters: a window into ligand-nanoparticle interactions. *Chemical Communications*.

[28] Nevers D. R., Williamson C. B., Savitzky B. H. (2018). Mesophase formation stabilizes high-purity magic-sized clusters. *Journal of the American Chemical Society*.

[29] Chen M., Luan C., Zhang M. (2020). Evolution of CdTe magic-size clusters with single absorption doublet assisted by adding small molecules during prenucleation. *Journal of Physical Chemistry Letters*.

[30] Sadeghi S., Abkenar S. K., Ow-Yang C. W., Nizamoglu S. (2019). Efficient white LEDs using liquid-state magic-sized CdSe quantum dots. *Scientific Reports*.

[31] Zhang J., Hao X., Rowell N. (2018). Individual pathways in the formation of magic-size clusters and conventional quantum dots. *Journal of Physical Chemistry Letters*.

[32] Luan C., Gokcinar O. O., Rowell N. (2018). Evolution of two types of CdTe magic-size clusters from a single induction period sample. *Journal of Physical Chemistry Letters*.

[33] Hsieh T.-E., Yang T.-W., Hsieh C.-Y. (2018). Unraveling the structure of magic-size (CdSe)13Cluster pairs. *Chemistry of Materials*.

[34] Li L., Zhang J., Zhang M. (2020). Fragmentation of magic-size cluster precursor compounds into ultrasmall CdS quantum dots with enhanced particle yield at low temperatures. *Angewandte Chemie*.

[35] Zhang J., Li L., Rowell N. (2019). One-step approach to single-ensemble CdS magic-size clusters with enhanced production yields. *Journal of Physical Chemistry Letters*.

[36] Landes C., Braun M., Burda C., El-Sayed M. A. (2001). Observation of large changes in the band gap absorption energy of small CdSe nanoparticles induced by the adsorption of a strong hole acceptor. *Nano Letters*.

[37] Young A. G., Green D. P., McQuillan A. J. (2006). Infrared spectroscopic studies of monothiol ligand adsorption on CdS nanocrystal films in aqueous solutions. *Langmuir*.

[38] Dager A., Uchida T., Maekawa T., Tachibana M. (2019). Synthesis and characterization of mono-disperse carbon quantum dots from fennel seeds: photoluminescence analysis using machine learning. *Scientific Reports*.

[39] Zhou Y., Chen J., Bakr O. M., Sun H.-T. (2018). Metal-doped Lead halide perovskites: synthesis, properties, and optoelectronic applications. *Chemistry of Materials*.

[40] Drisdell W. S., Leppert L., Sutter-Fella C. M. (2017). Determining atomic-scale structure and composition of organo-lead halide perovskites by combining high-resolution X-ray absorption spectroscopy and first-principles calculations. *ACS Energy Letters*.

[41] Wheeler L. M., Sanehira E. M., Marshall A. R. (2018). Targeted ligand-exchange chemistry on cesium lead halide perovskite quantum dots for high-efficiency photovoltaics. *Journal of the American Chemical Society*.

