# Supplementary material for: Interplay between Perovskite Magic-Sized Clusters and Amino Lead Halide Molecular Clusters
Source: Research (Wash D C). 2021 Jan 7;2021:6047971. doi: 10.34133/2021/6047971 (PMC7877386; doi:10.34133/2021/6047971)
Supplement: Supplementary Materials — Figure S1: PbX2-BTYA FT-IR spectra. Figure S2: conversion of MABr+PbBr2 + VA + BTYA MCs back to PMSCs. [file 6047971.f1.docx]

Supporting Information

Interplay between Perovskite Magic Sized Clusters and Amino Lead Halide Molecular Clusters

Evan T. Vickers,^†^ Ziyi Chen,^‡^ Vivien Cherrette,^†^ Tyler Smart, ^†^ Peng Zhang,^‡^ Yuan Ping,^†^

and Jin Z. Zhang*^,†^

*^†^Department of Chemistry and Biochemistry, University of California, Santa Cruz, CA 95064, USA*

*^‡^Department of Chemistry, Dalhousie University, NS, Canada B3H 4R2*

AUTHOR INFORMATION

Corresponding Author: zhang@ucsc.edu

**Experimental Methods**

**Materials**

*N,N*-Dimethylformamide (DMF) 99.5% Fisher Scientific. Toluene 100% LabChem Inc. Butylamine (BTYA) 99% Oakwood Chemical. Valeric acid (VA) 99% Alfa Aesar. Methylammonium chloride (MACl), methylammonium bromide (MABr), methylammonium iodide (MAI), lead chloride (PbCl_2_), lead bromide (PbBr_2_), and lead iodide (PbI_2_) GreatCell Solar.

**Spectroscopy**

Absorption spectra were measured with an Agilent Technologies Cary 60 UV-Vis

spectrophotometer. Fluorescence spectra were measured using a HORIBA Jobin Yvon

Fluoromax-3 spectrofluorometer and all samples were excited at 365 nm. Fourier transform-infrared (FT-IR) spectra were obtained using a PerkinElmer Spectrum One FT-IR spectrometer. FT-IR spectra of PbX_2_-BTYA samples are shown in **Figure S1.** Fourier transform extended X-ray absorption fine structure (FT-EXAFS) were measured at Sector 9-BM beamline at the Advanced Photon Source (operating at 7.0 GeV) in Argonne National Labs, Chicago, IL. The solid samples were packed in a Kapton pouch. The end station was equipped with a double-crystal Si(111) monochromator for wavelength selection. All samples were measured in fluorescence mode at room temperature. The fittings consist of an amplitude reduction factor of 0.9, *k* weight of 3, *k* range for Fourier transform 2-10 Å^-1^, and *r* range 1.6-3.6 Å. Quantum calculations were performed using the FEFF6 program to obtain the theoretical scattering amplitudes and phase-shift functions to fit the scattering paths with the Artemis program.

**PbX_2_+BTYA Synthesis**


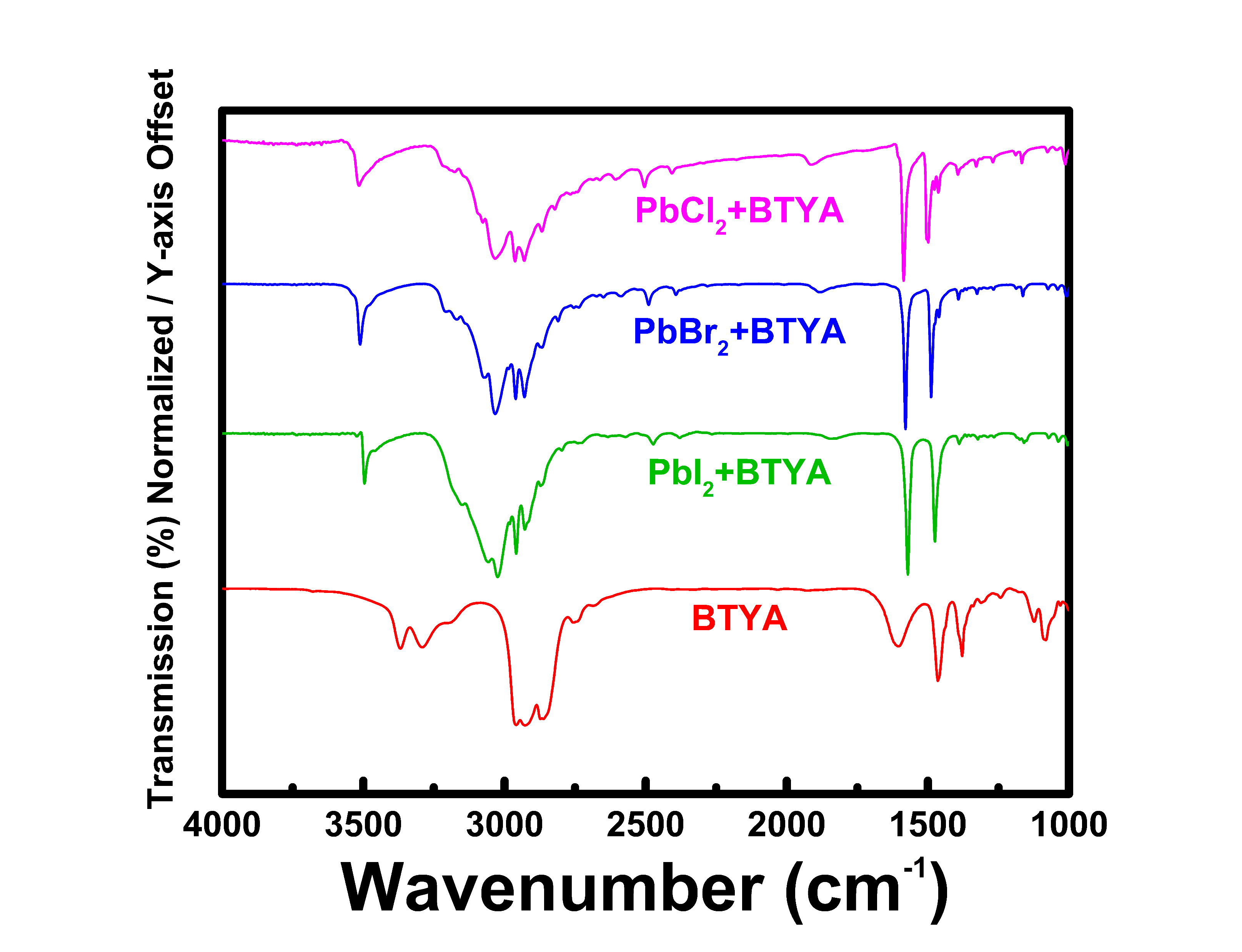


**Figure S1.** PbX_2_-BTYA FT-IR spectra.

For PbX_2_+BTYA samples, 0.20 mmol PbX_2_ is dissolved in 400 μL DMF. Then, 2.02 mmol BTYA is added and dissolved. For PbCl_2_ heat is required to dissolve. Next, 100 μL of this precursor solution is injected quickly into 5.0 mL toluene stirring at 1300 rpm. To wash the samples, the precipitate was broken up by vortex and waited until precipitate fell to the bottom of the vial. The solution was decanted of toluene and another 5.0 mL toluene was added. This was repeated two more times. For the PbI_2_+BTYA, the solution is not washed immediately after synthesis. After the synthesis, the solution is left in the dark for seven days. After seven days PbI_2_+BTYA is washed following the above method.

**PbX_2_+VA+BTYA Synthesis**

For PbX_2_+VA+BTYA samples, 0.20 mmol PbX_2_ is dissolved in 400 μL DMF. Then, 0.73 mmol VA is added and mixed by vortex. Next, 2.02 mmol BTYA is added and mixed by vortex. Finally, 100 μL of this precursor solution is injected quickly into 5.0 mL toluene stirring at 1300 rpm. The initial solutions are colorless and transparent and there is no evidence of precipitate or MSCs from optical measurements. However, for PbCl_2_+VA+BTYA and PbBr_2_+VA+BTYA precipitate starts to form after a 1:1 dilution or greater in toluene, and after waiting 72 hours. For PbI_2_+VA+BTYA, precipitate never forms and does not produce MSCs but produces QDs after 24 hours and diluting the solution 1:1 or greater in toluene.

**MAX+PbX_2_+VA+BTYA Synthesis**

For MAX+PbX_2_+VA+BTYA samples, 0.20 mmol PbX_2_ and 80 μmol MAX is dissolved in 400 μL DMF. Then, 0.73 mmol VA is added and mixed by vortex. Next, 2.02 mmol BTYA is added and mixed by vortex. Finally, 100 μL of this precursor solution is injected quickly into 5.0 mL toluene stirring at 1300 rpm. The initial solutions are colorless and transparent and there is no evidence of precipitate or MSCs from optical measurements. However, for MACl+PbCl_2_+VA+BTYA and MABr+PbBr_2_+VA+BTYA precipitate starts to form after a 1:1 dilution or greater in toluene, and after waiting 72 hours. For PbI_2_+VA+BTYA, precipitate never forms and does not produce MSCs but produces QDs after 24 hours and diluting the solution 1:1 or greater in toluene.

**MABr+PbBr_2_+VA+BTYA MC Back to PMSC**

After 4.3 h, an additional 9.0 mg of MABr was added to the undiluted solution. The solution was then sonicated for 1 min for mixing. The temperature of the solution was raised to 100°C, 55°C, 30°C, 19°C, and then 15°C, and the UV-vis absorption spectra was measured at each temperature, as shown in **Figure S2**.

**Figure S2**. Conversion of MABr+PbBr_2_+VA+BTYA MCs back to PMSCs.
